# The Biological Control of the Malaria Vector

**DOI:** 10.3390/toxins4090748

**Published:** 2012-09-19

**Authors:** Layla Kamareddine

**Affiliations:** Department of Biology, American University of Beirut, Bliss Street, Beirut 11072020, Lebanon; Email: lyk06@aub.edu.lb; Tel.: +961-135-0000; Fax: +961-174-4461

**Keywords:** malaria, *Plasmodium*, *Anopheles*, drug and insecticide resistance, health, environmental, and ecological side effects, alternative tools, biological control

## Abstract

The call for malaria control, over the last century, marked a new epoch in the history of this disease. Many control strategies targeting either the *Plasmodium* parasite or the *Anopheles* vector were shown to be effective. Yet, the emergence of drug resistant parasites and insecticide resistant mosquito strains, along with numerous health, environmental, and ecological side effects of many chemical agents, highlighted the need to develop alternative tools that either complement or substitute conventional malaria control approaches. The use of biological means is considered a fundamental part of the recently launched malaria eradication program and has so far shown promising results, although this approach is still in its infancy. This review presents an overview of the most promising biological control tools for malaria eradication, namely fungi, bacteria, larvivorous fish, parasites, viruses and nematodes.

## 1. Introduction

Malaria is one of the most common vector-borne diseases prevalent in tropical and subtropical areas of the world, including regions in Africa, Asia and America [1]. In 2010, over 1.2 million global malaria deaths were reported in both children and adults [2]. Malaria is caused by the protozoan parasites, belonging to the genus *Plasmodium*, residing in some female mosquitoes of the genus *Anopheles*. Among the 460 identified *Anopheles* species, 100 are reported as malaria vectors, and only 30–40 species of those reported vectors commonly transmit *Plasmodium* parasites [3]. Of all *Plasmodia*, only *P. malariae*, *P. ovale*, *P. falciparum*, *P. vivax* [4] and *P. knowlesi* [5] infect humans. Despite the numerous established findings that explain the process of the parasite propagation within the *Anopheles*, this vector borne disease remains one of the major health threatening problems world-wide. Eradicating malaria by targeting the *Anopheles* vector [6] using insecticide-treated nets (ITNs), long lasting insecticidal material (LMs), indoor residual spraying (IRS), and space spraying, along with proper preventive measures [7], was among the most important achieved strategies in the past years. For a period of two decades, the use of insecticides in controlling vector borne diseases, including malaria, was among the most reliable methods. Many compounds like mercuric chloride, Paris Green, phenols and cresols, naphthalene, Bordeaux mixture, rosin-fish oil soap, calcium arsenate, and nicotine sulfate, were used as conventional pesticides [8]. In the twentieth century, dichlorodiphenyltrichloroethane (DDT), the first synthetic organic insecticide, introduced a new epoch of vector control [9]. The use of IRS containing DDT and other chemicals in adult female *Anopheles* control showed great success [10,11,12,13,14]. IRS resulted in a drastic decrease in the recorded annual parasite index (API) in various regions of the world, a fact that drove the World Health Assembly to implement this approach in the 1955 malaria control strategy [15]. Also, there were many attempts to chemically control malaria by particularly targeting *Anopheles* at the larval stages. Paris Green (Copper Acetoarsenite) [16] and petroleum oils [17] were among the most successfully used chemicals in larval control. Although the widespread use of insecticide applications contributed to *Anopheles* control in various regions of the world, most of these applications, especially those relying on DDT usage, bypassed several important environmental and ecological considerations. As such, the environmental protection agency (EPA) prohibited the use of DDT in 1972 [18]. In 2001, the Stockholm Convention on persistent organic pollutants (POPs) also listed DDT as one of the twelve identified POPs [18]. Though epidemiological studies gave no evidence of the direct effect of DDT on inducing breast, liver, and pancreatic cancer, the ability of DDT to reside in many human tissues and cause various health related disorders, including problems in the liver, kidney, nervous, immune and reproductive systems, was another important reason to reconsider the use of such chemical compounds in malaria control [18]. Likewise, apart from being highly potent and cheap [18], the presence of toxic arsenic compounds in the chemical makeup of Paris Green was the major reason behind reassessing its role as a larvicide [18]. Several other larvicides including synthetic pyrethroids [19,20,21] and many organophosphates [22] are also rarely used these days. Though very effective, synthetic pyrethroids are extremely toxic to aquatic non-target organisms, mainly fish [23]. The remarkable toxic and persistent effects of many chemical applied insecticides were not the only obstacles facing the chemical control of malaria. The emergence of insecticide resistant mosquito strains [24] was another major impediment in such control strategies. These outgrowing strains drove the World Health Assembly resolution (WHA) to call for adopting and developing alternative approaches in controlling vector-borne diseases, thus decreasing the usage of insecticides. Integrated vector management (IVM) efforts are now oriented towards controlling *Anopheles* either at the larval stages and/or at the adult stages using means of biological control, where various concerns at the ecological, environmental, social, and economical levels are highly considered [25]. The use of biological agents shows no environmental contamination or *Anopheles* resistance. Their side effects on living beings including humans, domestic animals and on wildlife are minimal, if not completely absent. The importance of biologically controlling the malaria vector also falls within the functional diversity of different biological control agents (Table 1). Besides, many currently employed approaches and future set plans are now focusing on the use of genetically engineered microorganisms to either block the development of the malaria parasite within the *Anopheles* vector [26], or target the vector itself [27]. The biological control of the malaria vector is now considered a fundamental part of the recently launched malaria eradication program.

**Table 1:** Mechanisms of action, modes of application, and several limitations of some biological control agents.

Biological Control Agent	Commonly Used Strain	Effect	Application	Limitation	Corresponding Reference
**Entomopathogenic fungi**	Coelomomyces Culicinomyces Beauveria Metarhizium Lagenidium Entomophthora	Upon direct contact with the mosquito external cuticle. Slow killing. Affect the mosquito feeding habits. Affect the mosquito behavior and fitness conditions. Elevate the mosquito immune response and promote the production of secondary metabolites in the haemolymph.	In outdoor attracting odor traps. On indoor house surfaces. On cotton pieces hanging from the ceilings, bed nets and curtains.	Rapid fungal infection is required shortly after the mosquito picks up the malaria parasite.	[26,27,28,29,30,31,32,33]
**Bacterial agents**	Bacillus thuringiensis Bacillus sphaericus acetic acid bacteria (genus *Asaia*) wMelPop strain of *Wolbachia *	Suppress late instars and outgrowing pupae. Destroy larval stomach by endotoxin-proteins production. Rapidly colonize the male reproductive system and female eggs of many mosquito vectors.	At larval stages. At large scales. Through vertical transmission from mother to offspring.	*Bti* infections show no residual persistence post application. Only few studies address the effect of different bacterial agents on malaria vectors. Most of these studies are only experimentally approached without any further practical applications. Some bacterial strains like *Wolbachia* were not found to naturally infect *Anopheles*. Efforts to stably colonize *wMelPop* strains in *A. gambiae *failed.	[34,35,36,37,38,39,40,41,42,43,44,45,46,47,48,49,50,51,52,53,54,55,56,57]
**Larvivorous fish**	Gambusia affinis Cyprinodontidae Cyprinus carpio Ctenopharyngodon idella *Tilapia* spp.*Catla catla * Labeo rohita Cirrhinus mrigala Aphanius dispar Aplocheilus blocki Poecilia reticulata	Reduce larval density.	At larval stages. At low doses. In restricted open field system away from applied fertilizers and pesticides.	Great variability at the level of efficacy. Negatively affects the native fauna when introduced in many habitats. Require appropriate aquatic environments with reduced aquatic vegetations.	[55,56,57,58,59,60,61,62,63,64,65,66,67,68,69,70,71]
**Microsporidian parasites**	Vavraia culicis Edhazardia aedis	Combinatorial effects on different mosquito epidemiological traits: Decrease larval survival rates, decrease the number of adult mosquitoes, affect adult longevity, abort parasite development in the mosquito, affect mosquito biting rates.	At both larval and adult stages.	Seems only efficient when the effects on different mosquito epidemiological traits are combined.	[72,73,74,75,76,77,78,79,80]
**Viruses**	Densonucleosis viruses or denso viruses (DNVs)	Alter the ability of the mosquito to house the malaria parasite. Transduce certain anti-*Plasmodium* genes or specific *Anopheles* toxins in mosquito cells. Reduce mosquito longevity.	At both larval and adult stages. In the micro-environment of the host. Through vertical transmission among mosquito generations.	Only limited numbers of studies address the effect of viruses on malaria vectors control.	[81,82]
**Nematodes**	Different strains *(like Romanomermis iyengari *and *Romanomermis culicivorax) *of the *Mermithidae *species	Interfere in the mosquito reproductive behavior causing biological castration. Reduce mosquito populations. Decrease the rates of malaria transmission.	Mainly at larval stages.	Little is known about the parasitic effect of nematodes at the adult stages of mosquitoes.	[83,84,85,86,87,88]

## 2. Means of Biological Control

### 2.1. Entomopathogenic Fungi

The use of entomopathogenic fungus, as an alternative method for malaria vector control, seems to be very promising. Fungal species belonging to the genera *Coelomomyces*, *Culicinomyces*, *Beauveria*, *Metarhizium*, *Lagenidium*, and *Entomophthora* were mostly considered when studying the role of fungus in vector disease control [28]. Unlike other infectious agents, fungus does not require host ingestion; external contact with the insect’s cuticle is all that is needed to promote an infection. This way of launching an infection is not only practical and easily applied in the field, but also resembles many currently used chemical insecticide delivering strategies. Fungal spores can be applied in outdoor attracting odor traps, on indoor house surfaces, on cotton pieces hanging from ceilings, bed nets, and curtains, and can persist for a couple of months on many of these surfaces [29,30,31]. The fact that fungal infections can either act alone or in synergy with various insecticides, including DDT, and is equally effective against both insecticide resistant and insecticide susceptible mosquitoes was another major reason behind incorporating fungus in integrated vector management or in insecticide-resistant management approaches [89,90]. Many studies showed that insecticide resistant *Anopheles gambiae* are significantly more susceptible to fungal infections than insecticide susceptible strains [89], and that fungal infections kill mosquitoes at slower rates as compared to the insecticide killing rates [91]. Suppressing insecticide resistant mosquitoes at faster rates compared to susceptible ones and within prolonged durations compared to insecticide treated ones will eventually remove all insecticide resistant genes from the mosquito population, allow insecticide susceptible strains to breed, keep the fungus “evolution proof”, and collectively result in insecticide resistance management, without further insecticide usage [31,92]. This approach is highly effective for two major reasons. Since the *Plasmodium* parasite requires 10–14 days to complete its life cycle within the mosquito, then there is no need for rapid killing of the vector. Besides, these slow killing rates would only result in minimal fungal resistance-selective pressure, even if any resistance would eventually develop [31,92]. Many laboratory-based bioassays also showed that the mortality rates of adult *Anopheles* infected with the malaria parasite is considerably higher when exposed to fungal spores, and reaches 100% in some cases, compared to those of *Anopheles*, either infected with fungus or parasites alone. This killing effect was shown to be exerted within 7–14 days post-exposure, depending on the fungal strain used, the mode of infection, and the dose applied [32,93] For practical application purposes, a small scale field study done in village houses in Tanzania showed that even relatively low doses of fungal application on small surface areas result in 34% mosquito infection and in 75% reduction in the entomological inoculation rates of infected mosquitoes [93]. Such studies show that even with the currently available technologies, entomopathogenic fungus can be feasibly and effectively used as a vector control biopesticide. 

By closely examining the fungal “pesticidal” properties, fungi were shown to exert negative effects on malaria transmission by altering the behavior and fitness conditions of the mosquito vectors, without decreasing their densities. It has also been shown that fungal pathogens influence the feeding habits of mosquitoes, affecting their survival [33]. Even the survival rates of malaria parasites within the mosquito were shown to be affected [32,33]. Although the mechanism of action of fungi as anti-malarial agents has not been clearly elucidated, many studies point to a role of fungi in disrupting the mosquito nutritional balance, elevating its immune response, and/or resulting in the production of secondary metabolites in its haemolymph [31]. 

Many laboratory groups are now developing transgenic fungi for better mosquito borne disease control. Such approaches are thought to be highly effective, very specific, exert negligible negative environmental impacts, and have relatively minimal effects on the parental wild-type mosquito strains [26]. Recently, it was shown that infecting mosquitoes with genetically engineered *Metarhizium*, designed to produce anti-malarial peptides, blocked the transmission of the malaria parasite from its vector. This approach overcomes the necessity of rapid field applied fungal infection shortly after the mosquito picks up the malaria parasite, and prevents any possibility of developing fungal resistant mosquito strains, since transgenic fungi only kill adult mosquitoes [26]. Yet, the use of genetically engineered fungus compared to field applied fungal biopesticides is still not favored. Many argue that such strategies exert high fitness costs on the transgenic organism, are practically more complicated, and comparatively difficult to handle as field released pathogens [26]. In some cases, relying on anti-malarial factors might result, in the long term, in malaria parasite resistance, regardless of the fact that some fungal strains, like *Metarhizium* for example, could express multiple transgenes with different modes of action [26]. 

Apart from the promising aspect of the use of entomopathogenic fungi in controlling malaria, many concerns have been raised. The emergence of mosquito-insecticide resistance to every chemical class [94] raises the possibility of mosquitoes evolving certain fungal resistant mechanisms [95,96]. Moreover, although little is known about the genetic variation in *Anopheles* fungal susceptibility, such variation exists in other mosquito strains as in *Drosophila melanogaster* [97] and in aphids [98,99]. Many environmental and behavioral aspects that affect mosquitoes could also contribute one day to the development of certain fungal resistant *Anopheles* strains [99,100,101]. Despite this, the use of fungal biopesticides is still considered promising due to a number of reasons. The fact that pathogenic fungi exert their effects at relatively late stages of the *Anopheles* life cycle is here an important consideration. In the context of evolution of ageing, it is well known that delayed life time mutations are subject to week selection because they usually confer fitness benefits at the end of reproduction [102,103]. So even if fungal resistance could develop, only weak selection for such resistance would occur. This way of reducing selective pressure could, in turn, be translated into additional decades of effective fungal biopesticide usage [31]. Besides, some argue that selection for resistance might not even exist if fungal-resistant mechanisms entail metabolic costs. If metabolic expenses were to be paid in return, then all individuals in the *Anopheles* population would have to pay the price for a benefit that is only experienced by a few [31]. The direct anti-malarial effect caused by fungal infections on sporozoites, and the considerably high mortality rates of fungal-treated parasite-infected mosquitoes compared to those lacking a parasitic infection also aids in overcoming the possibility that fungal biopesticides would be undermined by any sort of mosquito resistance. It is, therefore, highly desirable to isolate fungal strains that can reduce sporozoite prevalence, without causing any mosquito death. Such direct pathogenic effect would reduce the fitness of only *Plasmodium* infected mosquitoes, circumventing any selection for fungal resistance in uninfected mosquitoes [32]. This might even result in selection for increased malaria refractoriness [104].

### 2.2. Bacterial Agents

The use of bacterial agents in controlling vector borne diseases has raised several concerns as to whether these microorganisms are highly effective, environmentally safe, non-toxic, and exert selective effects. Among the many tested bacteria, *Bacillus thuringiensis* (*Bti*) and *Bacillus sphaericus* (*Bs*) are the most promising bacterial larvicidal strains in malaria vector control [34,45]. *Bacillus* strains are cheap, can be locally manufactured, easily handled, and practically applied [105]. Compared to chemical insecticides, *Bti* and *Bs* showed faster spreading abilities. Within five years of their discovery, these bacterial strains rapidly colonized Europe and Africa, and methodically participated in routinely applied large-scale mosquito control operations in these regions [36,37]. *Bti* is now thought of as an alternative approach to synthetic chemical insecticides, since its association with resistant mosquito strains and environmental crisis is comparably insignificant [105].

The need of integrated microbial larvicide mosquito control strategies is today highly considered in many countries in the tropics. In South America for example, considerable efforts are being made in testing new local bacterial strains, their formulations [106,107,108] and the possibility of combining such approaches with others that target mosquitoes at the adult stages [109,110,111,112,113]. Although only few studies were done to test the effect of *Bti/Bs* on African malaria vectors [38,39,40,41,42,43,44], and although these studies were more of experimental rather than large-scale practical application [45,46], their established results showed effective roles of these *Bacillus* strains, but highlighted the need for additional work at this particular level, along with broader disseminations and practical implications [105]. Opposing many of the suggestions [34,114], these recorded data showed that the larvae of *A*. *gambiae* are highly sensitive to *Bti* and *Bs* infections compared to the larvae of other mosquito species like *Aedes*, *Culex quinquefasciatus*, and *A. arabiensis* [43,105,115]. Under laboratory conditions, the *A. gambiae* larvae were further publicized to be more susceptible to *Bs* infections than to *Bti* infections [105]. Open field trials also showed that only low dosages of *Bti* infections are enough to effectively suppress late instars and out growing *Anopheles* pupae [105]. The importance of using low dosage formulations is highly valued since it keeps operational costs low, especially if the microbial infections were to be applied on a weekly basis [105]. In such studies, the presence or absence of residual activity has to be also taken into account when evaluating the effect of bacterial infections on the larval populations. *Bti* infections showed no residual persistence post application [47]. A study done on *Bti* infected larval populations in the Democratic Republic of Congo revealed that infected larvae start recovering 5–7 days post treatment at the latest [39].On the other hand, *Bs* infections were shown to result in great residual larvicidal activities. *Bs* bacterial spores persisted for a long period in the environment and were recycled in the larval guts after dying [116]. Detecting residual persistence has to be associated, in turn, with a number of factors including the method of application, the formulation used, and the specific larval species and its density [105]. High density larvae added at regular intervals showed longer residual activities post *Bs* applications [117]. At the level of practical applications, larvicide formulations drawn from the H-14 serotype of *B. thuringiensis* are now being used in vector disease control, and those of the 1593 of *B. sphaericuss* strain will soon reach the market. 

For even less costly and better control strategies, and since the toxicity of *Bti* and *Bs* mainly resides in the production of endotoxin proteins that destroy the larval stomach and cause death, many genetic engineering techniques are now oriented towards cloning several genes encoding many *Bti* and *Bs* endotoxin proteins, thereby generating new recombinant bacterial strains. The detected effectiveness of some newly emerging bacterial strains was 10 times more than that of either *Bti* or *Bs* active ingredients alone [118,119]. The most effective recombinant produced was the one containing almost all *Bti* toxins, including Cry4A, Cry4B, Cry11A, and Cyt1A, combined with the binary (Bin) endotoxin of the *Bs* species [120]. Interestingly, the Cyt1A endotoxin protein, synergized with the Cry endotoxin proteins, not only delays resistance to Cry proteins and enables long term usage, but also allows *Bs* resistance to be overcome, and broadens the spectrum of activity of these endotoxins to reach many important disease vectors and nuisance species including *A. gambiae*, *A. arabiensis*, *Culex*, *Ochlerotatus*, and *A. aegypti* [118,119]. Many groups also suggested cloning some genes of newly discovered mosquitocidal proteins like the Mtx proteins [121] and some peptides such as the trypsin-modulating oostatic factor [120] that could be feasibly engineered and highly expressed in recombinant bacteria [118]. 

The use of mosquito-bacterial symbionts, that are vertically transmitted and widespread among mosquito populations, is another recently suggested approach for vector-borne disease control. Promising candidates are so far acetic acid bacteria of the genus *Asaia* which were found to colonize the male reproductive system and female eggs of several human vectors including *A. aegypti*, *A. gambiae*, *A. stephensi*, and *A. Albopictus*, and which undergo vertical transmission from mother to offspring, thereby rapidly colonizing the mosquito populations [51,52,53,54,55]. The maternally inherited, endosymbiont wMelPop strain of *Wolbachia* is another interesting bacterial candidate which when introduced into *A. aegypti* resulted in an up regulation of the mosquito immunity and reduced its life span, inhibiting the development of filarial nematodes in these mosquitoes [53]. While wMelPop can efficiently colonize *A. aegypti* mosquitoes through maternal inheritance, efforts to stably colonize *A. gambiae* mosquitoes with *Wolbachia* have failed so far, and anophelines seem to be naturally uninfected with this bacterium. Nevertheless, the transient somatic infection of *A. gambiae* with two diverse *Wolbachia* strains significantly reduced *P. falciaprum* oocyst levels in these mosquitoes [54]. In short, the use of microbial agents is now highly considered in combating malaria. These agents either directly target the *Anopheles* vector itself, or abort the development of the *Plasmodium* parasite within the mosquito.

### 2.3. Larvivorous Fish

The use of predatory fish that feed on mosquito larvae was one of the old suggested methods for controlling vector diseases at the larval stages. Prior to the 1970s, mosquito control by means of fresh water *Gambusia affinis* predominated. These native southeastern United States species were widely introduced around the world for mosquito control [55]. Other fish species, like those belonging to the family *Cyprinodontidae*, were also copiously used, for at least 100 years, in larval control [56]. As compared to chemical agents, larvivorous fish were shown to be more effective. They can be used at low doses, are harmless to both humans and wildlife, cheap to produce in most cases, and exhibit minimal risks of mosquito resistance [57]. Although promising, the use of larvivorous fish as a means of vector control agent was questioned with time. Introducing new fish species into certain aquatic environments showed great variability at the level of efficacy and exerted many negative impacts on the native fauna where these fish were brought in [58]. The introduction of *Gambusia* in certain habitats, for example, resulted in the elimination of many native fish species from these habitats [59]. Therefore, to minimize the loss of native species and reduce the variability in effectiveness of larval control among different aquatic environments, many pre-application studies were done to establish the most suitable fish-habitat model. Most of these studies related the efficacy of larvivorous fish to two major factors. The first includes the amount of larvae eaten by fish in different water bodies, and the second is mainly associated with the appropriate conditions of the aquatic environment where new fish species are introduced [55].Aquatic vegetation strongly affects the first factor. The effects, in such a case, may be interpreted at the level of both the fish and the mosquito larvae. When aquatic vegetation interferes with the feeding habits of the fish, it, indirectly, protects the larvae from their predators. Therefore, periodic vegetation removal is needed to facilitate the activity of the fish and make this approach effective [60]. As for a suitable aquatic environment, finding native larvivorous fish species dwelling within the same mosquito breeding sites is highly favored over changing the mosquito breeding sites to fit with the environment of the fish [61]. Rice fields, away from any sort of applied pesticides or fertilizers that negatively affect fish stocks in these watered fields, were shown to be the most suitable open field system to harbor larvivorous fish [62]. Many studies showed that fish are also highly effective when the mosquito breeding sites are restricted in number and are well defined. In China, for example, the presence of carp fish in certain rice fields, reduced the number of malaria cases, and improved rice yield fish production in that country [58].

Challenging *A. sinensis* with a mixed population of *Cyprinus carpio*, *Ctenopharyngodon idella* and *Tilapia* spp. resulted in a significant reduction in the anopheline larval density [58]. Other studies also showed that challenging different *Anopheles* species with a mixed population of *Cyprinus carpio*, *Ctenopharyngodon idella*, *Catla catla*, *Labeo rohita*, and *Cirrhinus mrigala* resulted in 81% reduction in their larval density [63]. Furthermore, introducing larvivorous fish into man-made water containing constructs in many urban and peri-urban areas in India and Africa showed promising results. The use of native *Aphanius dispar*, for example, caused a 97% and 95% reduction in the larval density of *A. culicifacies* and *A. adanesis*, respectively [64]. Similarly, introducing *Gambusia affinis* into water wells resulted in 98% reduction in the larval density of *A. stephensi* [65]. Other *Anopheles* species including *A. gambiae* and *A. subpictus* also showed significant susceptibility to either native or foreign larvivorous fish species like *Aplocheilus blocki*, and *Poecilia reticulate* [66,67,68,69]. A study conducted in a number of riverbed pools located below many major dams in Sri Lanka also showed the potential of *Poecilia reticulate* in anopheline control [70]. Interestingly, combining native *Aplocheilus blocki* in water tanks or in any other mosquito breeding site with *Bti* strains in smaller habitats not only resulted in a significant reduction in the *Anopheles* larval density, but was also more effective in reducing the annual malaria parasite index in these infected mosquitoes as compared to those treated with conventional insecticide sprays [55].

Many countries like Greece, Italy, Georgia, Spain, India, Malaysia, Madagascar, and Papua New Guinea have heavily relied on larvivorous fish as a major strategy in malaria vector management [16]. Although reducing adult *Anopheles* is considerably effective, some argue that such an approach might, under certain conditions, suppress the local mosquito vector population [122,123,124]. Also, targeting anopheline larvae instead of adults was reconsidered for many other reasons [125,126]. Larvae, for instance, unlike adults, cannot easily avoid control measures by escaping from their breeding sites [127]. Larval control was shown to be highly valuable in areas like Eritrea where *Anopheles* are exophilic and/or bite people before going to bed, defeating the effectiveness of using indoor residual sprays and impregnated bed nets [71]. 

### 2.4. Other Biological Control Agents

Other biological control agents include the use of parasites, viruses and nematodes in controlling the malaria vector. Evaluating the effectiveness of these approaches is based on two major criteria. It is how efficient the control agent can be in substantially decreasing the rate of vector transmission and to what extent can this tool be evolutionary sustainable. Relying on certain parasites like *Vavraia culicis* and *Edhazardia aedis* to abort the development of other parasite species like *Plasmodium*, or to target the mosquito vector itself, might seem somehow peculiar. Recent studies have shown promising roles of microsporidian parasites in malaria control. The effectiveness of these parasites falls within their ability to exert combinatorial effects on several important epidemiological traits of the mosquito. Microsporidians moderately decrease the larval survival rates, thereby decreasing the number of adult mosquitoes [72]. They also, moderately, affect the adult longevity [73], the development of the malaria parasites in the mosquito [74,75,76,77,78], and the biting rates of the mosquito vector [79]. Although only moderate, when combined, these affected traits result in a considerable reduction in the intensity of malaria transmission. If the 25% recorded increase in the larval mortality rates post microsporidian parasitic infection were added to the 20% increase in the adult mortality rates and to the 25% reduction in mosquito infectivity, along with a significant reduction in the biting rates of infected mosquitoes, then the overall malaria transmission process would be lowered by 80% [80]. 

Although many questions have been raised as to whether the intense use of microsporidia in malaria vector control would eventually result in the evolution of microsporidian-resistant larvae, this evolutionary process does not seem to completely eliminate the role of microsporidia in *Anopheles* control. Several groups suggest an inverse genetic correlation between the larval parasitic tolerance and their adult longevity. They argue that the ability of mosquitoes to gain tolerance to the microsporidia parasites is, in turn, compensated for by a decline in their life span and biting habits [80,128]. If this suggestion could be experimentally proven, then the development of resistant larval strains would be evolutionary costly to the malaria vector and indirectly contribute to its eradication [80]. 

Many gaps still exist in our understanding of the key molecular interactions between the parasite and its vector. If such interactions were better understood, many paratransgenic approaches that genetically modify symbiotic microbes to express different effector molecules would be further developed, reducing the longevity of the mosquito and antagonizing the development or transmission of the malaria parasite [50,129]. A suitable microbial candidate for this purpose should fulfill a number of requirements. These requirements include the ability of the microbe to be readily propagated and stably engineered to express certain genes of interest without causing any fitness cost on the mosquito, exhibit a parasitic, commensal, or mutualistic relation with its host, and be easily transported into wild type mosquito populations [129]. Ideally, the engineered microbe should also have the ability to be sustained in its host microenvironment with minimal, if any, negative impact on different non-target species [81]. The first identified candidates to perform this task were the Densonucleosis viruses, or “denso viruses” (DNVs), which belong to the Parvoviridae family of viruses that are known to infect arthropods, including mosquitoes [82]. The *A. gambiae* denso virus (AgDNV) was shown to be highly infectious to *Anopheles* at larval stages. AgDNV was also shown to be able to circulate in adult mosquito tissues and undergo vertical transmission between generations [81]. The use of AgDNV is now highly considered in malaria control strategies since these recombinant viruses were able to transduce the expression of an exogenous gene (EGFP) in mosquito cells. Mosquitoes infected with EGFP-transducing virions not only expressed EGFP in epidemiologically relevant tissues but were also genetically transmitted to their offspring in a very similar manner to that of wild type viruses [81]. Therefore, the important roles of these viruses lie in their ability to transduce certain anti-*Plasmodium* genes or *Anopheles* specific toxins in mosquito cells, in addition to the feasibility of using such a control system for transient gene expression and RNAi based laboratory research [81].

The use of elongated round-headed nematode worms, like *Mermithidae*, is also among the list of suggested biological agents in malaria control. About twenty five different *Mermithidae* worm species were found to dwell at the larval stages of different mosquito strains [83]. Very little is known about the parasitic effect of nematodes at adult stages. Only few studies have shown that nematodes negatively affect many adult mosquito species including *Aedes* [130,131], *Ochlerotatus* [83,130,132], *A. punctipennis* [84], *Coquillettidia perturbans* [131], and *A. letifer* [133]. While studying malaria at the entomological level, Vythilingam, Krishnasamy, Chen, and their group members also detected the presence of *Mermithid* parasites in three different adult *Anopheles* species [133]. Despite the fact that *Mermithids* do not directly inhibit the blood feeding behavior of mosquitoes, their effect lies with their ability to interfere in the mosquito’s reproductive system, resulting in biological castration [85,86]. In the long term, these parasitic nematodes will eventually result in a drastic reduction of the mosquito populations and in a considerable decrease in the malaria transmission rates. A study done in Pochutla, Oaxaca, Mexico, an endemic area of malaria, showed that *Romanomermis iyengari*, one strain of the *Mermithid* species, is very useful in the larval control of *A. pseudopunctipennis* [87]. The continuous application of around 3000 *Romanomermis iyengari* per meter square, on a 30,000 meter square area of *A. pseudopunctipennis* breeding sites, for a period of nine months, resulted in 46% to 100% decrease in the infection rates of the malaria parasite, and in a 38.1% to 99.8% reduction in the *Anopheles* larvae [87]. *Romanomermis iyengari* was also shown to recycle and persist for five months in some mosquito breeding sites [87]. Introducing *Romanomermis culicivorax*, another strain of the *Mermithid* species, in certain *A. albimanus* larval habitats in Colombia also showed considerable abilities of this parasitic worm to establish itself in these areas, recycle within 27 months, reduce the *A. albimanus* larval population, and result in a progressive decrease in malaria transmission, mainly among school children [88]. The use of parasitic nematodes in malaria vector control is not only effective in reducing malaria transmission among humans living in the *Anopheles* breeding sites, but also among those dwelling in nearby regions [87].

## 3. Conclusion

To date, many strategies have been used in malaria control. These strategies either abort the development of the *Plasmodium* parasite within the mosquito, or suppress the mosquito vector itself. Nevertheless, many factors such as relying on ineffective conventional vector control approaches, shortage of epidemiological control basis, scarce availability of resources and infrastructure, and poor management plans lead to a decline in the effectiveness of controlling malaria at the level of its vector [18,134]. Failure of mosquito control was also a result of environmental variations and changes in the behavioral features of many mosquito species like the emergence of insecticide resistant mosquito strains [18,134]. Taken together, these consequences highlighted the need of alternative vector control strategies. Shifting towards biological control of *Anopheles* was mainly due to its negligible side effects on humans, wild-life, and on the environment, in addition to the very minimal recorded cases of mosquito resistant strains to these biological agents. Although promising, the use of biological means in the recently launched malaria eradication program is still in its infancy. Understanding the exact mechanisms of the mosquito-pathogen interaction should be the focus of future research.
